# The microRNAs in an Ancient Protist Repress the Variant-Specific Surface Protein Expression by Targeting the Entire Coding Sequence

**DOI:** 10.1371/journal.ppat.1003791

**Published:** 2014-02-27

**Authors:** Ashesh A. Saraiya, Wei Li, Jesse Wu, Chuan H. Chang, Ching C. Wang

**Affiliations:** 1 Department of Pharmaceutical Chemistry, University of California-San Francisco, San Francisco, California, United States of America; 2 Institute for Biomedical Informatics, National Yang-Ming University, Taipei, Taiwan; University of California Los Angeles, United States of America

## Abstract

microRNAs (miRNA) have been detected in the deeply branched protist, *Giardia lamblia*, and shown to repress expression of the family of variant-specific surface proteins (VSPs), only one of which is expressed in *Giardia* trophozoite at a given time. Three next-generation sequencing libraries of *Giardia* Argonaute-associated small RNAs were constructed and analyzed. Analysis of the libraries identified a total of 99 new putative miRNAs with a size primarily in the 26 nt range similar to the size previously predicted by the *Giardia* Dicer crystal structure and identified by our own studies. Bioinformatic analysis identified multiple putative miRNA target sites in the mRNAs of all 73 VSPs. The effect of miRNA target sites within a defined 3′-region were tested on two *vsp* mRNAs. All the miRNAs showed partial repression of the corresponding *vsp* expression and were additive when the targeting sites were separately located. But the combined repression still falls short of 100%. Two other relatively short *vsp* mRNAs with 15 and 11 putative miRNA target sites identified throughout their ORFs were tested with their corresponding miRNAs. The results indicate that; (1) near 100% repression of *vsp* mRNA expression can be achieved through the combined action of multiple miRNAs on target sites located throughout the ORF; (2) the miRNA machinery could be instrumental in repressing the expression of *vsp* genes in *Giardia*; (3) this is the first time that all the miRNA target sites in the entire ORF of a mRNA have been tested and shown to be functional.

## Author Summary

*Giardia lamblia* is a protozoan parasite causing the diarrheal disease giardiasis. Variant-specific surface proteins (VSP) in *Giardia* are likely involved in its evasion of host immune response. Their expression is regulated by microRNAs (miRNA). To determine the full complement of miRNAs in *Giardia*, three cDNA libraries of *Giardia* Argonaute associated small RNAs were constructed and analyzed to identify a total of 105 miRNAs. Bioinformatic target identification showed that 102 of the 105 miRNAs find their putative target sites in *vsp* mRNAs. When only the target sites within the 3′ region,100 nts upstream of the stop codon, were tested against their corresponding miRNAs, however, only partial repression of VSP expression was observed. When all the miRNA target sites in the open reading frames of *vsp* mRNAs were examined, however, they all turned out to be functional. A saturation of them with the corresponding miRNAs resulted in a full repression of VSP expression, suggesting that this is the mechanism of miRNA repression of VSP expression in *Giardia*. The ability of miRNAs to regulate target sites throughout the entire open reading frame also provides the first indication that all the miRNA target sites in an mRNA are functional.

## Introduction

The deeply branching protozoan parasite *Giardia lamblia*, is responsible for the diarrheal disease giardiasis, which affects over 200 million people worldwide [1], [2]. In developing countries, multiple exposures to the pathogen result in repeated chronic infections. The persistence of infection is believed to be due to variations of the variant-specific surface proteins (VSPs) on the membrane surface of *Giardia* trophozoites. Only a single species of VSP is expressed on the membrane surface of a trophozoite at a given time, which is replaced by another VSP every 6 to 13 generations [3], [4], [5]. This variation likely allows *Giardia* to avoid the host immune response during infection [6]. VSPs are zinc finger-containing proteins containing two well-conserved structural motifs localized to the C-terminal domain of the molecule [7]. Motif 1, located upstream from the C-terminus, contains the zinc finger domain. Motif 2, located at the very C-terminus, is likely the transmembrane domain, which is followed by a 5 amino acid sequence CRGKA with a yet unknown function [8], [9]. Using these structural criteria, it was recently estimated that there are about 73 *vsp* genes in *Giardia* WB isolate [7]. Although it might not be the final number of *vsps* upon further investigation in the future, it is, nevertheless, the best verified number at present time. We have thus used it in the present study.

The regulatory mechanisms of VSP expression and variation have been poorly understood. All the *vsp* mRNAs appear to be continuously transcribed in *Giardia* trophozoites [10], [11]. Regulation of *vsp* expression is thus most likely at the posttranscription level. Our previous studies have postulated the involvement of a translational-repression mechanism involving a microRNA (miRNA) machinery in regulating VSP expression [12], [13], [14], [15], [16]. Six miRNAs have since been identified in *Giardia*, among which, five were found to translationally repress the expression of specific *vsp* mRNA subsets in *Giardia*, though full repression has not yet been observed (see below) [12], [13], [14], [15], [16].

miRNAs are small regulatory RNAs responsible for post-transcriptional regulation of mRNA expression among eukaryotes [17]. A primary miRNA in the nucleus is first processed by the nuclear RNaseIII, Drosha/Pasha, to generate a hairpin shaped pre-miRNA, which is then transported into the cytoplasm where another RNaseIII, Dicer, further processes it into a double-stranded miRNA [17]. The double-stranded miRNA is bound to an Argonaute protein, which releases its passenger strand and forms the miRNA-induced silencing complex (miRISC) [17]. Interaction between the miRISC-associated miRNA and its mRNA target site results in translational repression and degradation of the mRNA [18], [19]. In higher eukaryotes, the majority of miRNA target sites are found in the 3′ untranslated regions (UTR) of mRNAs [20], [21], though a few target sites have also been identified in the 3′-end of the coding region [22], [23], [24], [25], [26], [27], [28], [29]. A further search for conserved regions in coding regions also revealed that let-7 miRNA targets the Dicer within its coding sequence [30]. A recent study further suggested that the reduced frequency of functional miRNA target sites in the coding region of an mRNA could be due to ribosomal displacement of the miRISC during translation resulting in reduced miRNA-mediated translational regulation and an evolutionary bias against coding region target sites [31]. This hypothesis may explain why the majority of target sites have been primarily found in the 3′ UTR ∼15 nts away from the stop codon [20], [21]. But up to 50% of miRNA target sites have been identified in the open reading frames (ORFs) with a few hits in the 5′ UTR from HITS-CLIP analysis [32], [33]. These target sites have not yet been vigorously tested for their potential functions. Recent results from the large-scale protein mis-expression studies suggested that the target sites in upstream ORFs are actually functional, and can enhance the miRNA regulation when a 3′ UTR target site is also present [34]. The biological significance in the miRNA target sites present in the upstream portion of ORF remains thus to be further clarified.

In *Giardia*, 6 miRNAs (miR2, miR3, miR4, miR5, miR6 and miR10) have been identified and characterized [12], [13], [14], [16]. Since the mRNAs in *Giardia* have 3′-UTRs with an average length of only 30 nts [35], [36], there is in some cases no miRNA target site in the 3′-UTR or only one spanning across the stop codon and shared by both 3′-UTR and the 3′-end of ORF. A more recent report by Franzen et al. (2013) indicated that the median 3′-UTR length is 80 nts [37]. The additional length of the 3′ UTR may contain more than one miRNA target site but has not yet been tested. When a defined region of 100 nts upstream and 50 nts downstream of the stop codon in mRNAs was examined, 5 of the 6 identified miRNAs (with the exception of miR3) were shown to have their putative target sites in that region among certain *vsp* mRNAs. All 5 miRNAs demonstrated partial repression of either a *Renilla* luciferase reporter gene containing the corresponding target site in its 3′ UTR or expression of the *vsp* mRNA carrying the miRNA target sites in the defined 3′-region. The effects were additive when the target sites were separated from each other. This observation raised a probable scenario for a miRNA-mediated mechanism of inhibiting expression of the *vsp* genes in a *Giardia* trophozoite. The *vsp* mRNAs may each carry multiple target sites, allowing for multiple miRNA actions. When all the sites are saturated with their corresponding miRNAs, expression of the mRNA may be completely repressed.

In order to verify this hypothesis, the complete spectrum of miRNAs in *Giardia* trophozoites needs to be identified. We analyzed 3 next-generation sequencing (NGS) libraries from *Giardia* Argonaute (GlAgo)-associated small RNAs and identified a total of 105 putative miRNAs, including the 6 previously characterized miRNAs. All the newly identified miRNAs tested thus far were able to partially repress expression of their corresponding *vsp* mRNAs. Only partial repression was achieved when only target sites confined within the artificially defined 3′-region were saturated with miRNAs. When most of the target sites in the ORF of a *vsp* mRNA were saturated with miRNAs, however, a near total repression of expression was observed.

## Results

### Small RNA library construction and sequence analysis

We have previously reported co-immunoprecipitation of tagged GlAgo with a well-defined small RNA band in the 26 nt range [14]. The small RNAs in this band contained a 5′ phosphate group and a 3′ hydroxyl group and could be primarily the miRNAs [14]. To sequence these small RNAs, we constructed 3 independent libraries for NGS, using the GlAgo associated small RNAs from *Giardia* WB trophozoites as templates [14]. Two independent libraries (TDIP1 & TDIP2; Trophozoite Detergent GlAgoIP) were constructed from the small RNAs recovered from GlAgo immunoprecipitation after detergent lysis of *Giardia* trophozoites. A third library (TDSIP; Trophozoite Detergent/Sonication GlAgoIP) was constructed from cells by the same procedure except that a sonication step was added to the detergent lysis to insure a more complete trophozoite breakdown. As the subsequent results will verify (see below), the added sonication did not significantly alter the experimental outcome.

The 3 libraries thus constructed were sequenced using the Illumina HiSeq 2500 sequencer and each produced over 190 million reads (Table 1). After removing the adaptors, low quality sequences (Q<20) and sequences smaller than 20 nts or larger than 32 nts, the 3 libraries each retained over 95% of the original reads (Table 1). Over 60% of the pre-processed reads in each of the remaining 3 libraries could be aligned to the genome using the Bowtie software [38] and *Giardia* genome scaffolds downloaded from GiardiaDB (Table 1) [39]. About 40% of the aligned reads were derived from tRNAs and rRNAs, which was increased to 50% when sonication was included in preparing the lysate. These sequences were removed, and the three libraries each retained an average of 2 million unique reads (Table 1).

**Table 1:** Identification of unique reads from three NGS libraries.

Libraries	Raw Reads	Preprocessed Reads	Aligned Reads	Unique Reads (Unique 5′ ends)
TDIP1	194,583,375	185,867,204 (95.52%)	120,406,398 (64.78%)	2,410,817 (1,813,868)
TDIP2	202,723,476	192,721,824 (95.07%)	119,977,042 (62.25%)	2,136,098 (1,611,696)
TDSIP	198,613,257	189,593,935 (95.46%)	138,362,871 (72.97%)	2,352,890 (1,785,218)

**Figure 1:**
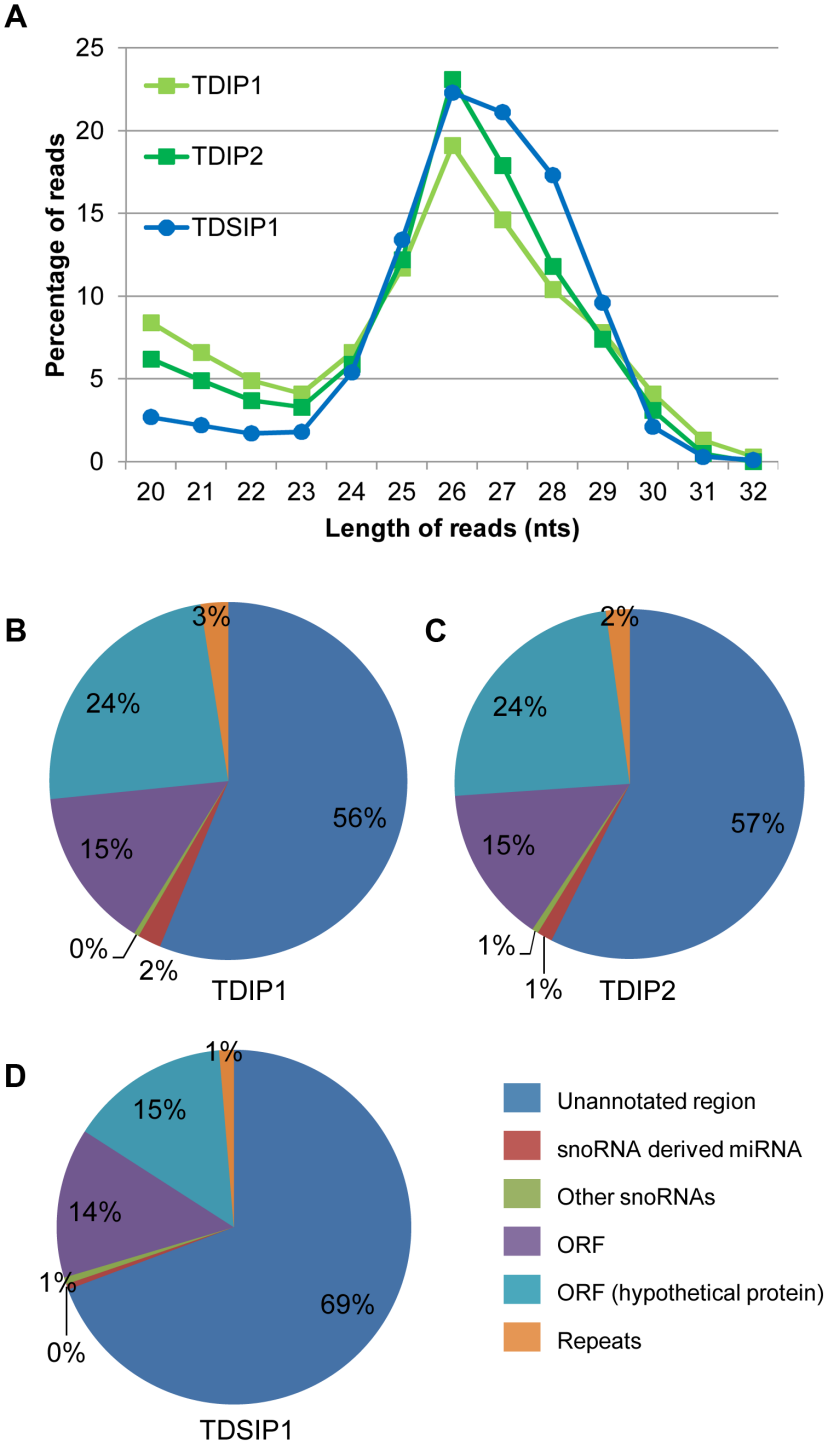
Analysis of the NGS libraries. **A**) Size distribution of sequences remaining after alignment to the *Giardia* genome and removal of rRNA and tRNA. A peak at 26 nts corresponds to the expected size of miRNAs in *Giardia*. **B**) Analysis of read origins in the TDIP1 library. The majority of potential miRNAs are derived from ORFs or unannonated regions. **C**) Analysis of read origins in the TDIP2 library. **D**) Analysis of read origins in the TDSIP library shows a 12–13% increase in the reads derived from unannonated regions with a corresponding decrease in reads derived from ORFs.

Length distributions of the unique reads among the three libraries are highly similar. There is a 26 nt peak, which agrees with the size of miRNAs anticipated from the *Giardia* Dicer (GlDcr) crystal structure [40] and the sizes of miRNAs identified in our previous studies [12], [13], [14], [15], [16] (Figure 1A).

The majority of sequences among the unique reads are derived from unannotated regions of the *Giardia* genome followed by sequences derived from the ORFs (Figures 1B, C, D). They constitute about 95% of the populations in all 3 libraries. The remaining sequences are derived from snoRNAs including primarily the few snoRNA-derived miRNAs we have already identified [12], [13], [15], [16]. The sequence distributions in the 3 libraries indicate that they are of high quality with data very similar to one another.

### Identification of putative miRNAs

Putative miRNA candidates in the 3 libraries were selected by initially determining the minimum read counts for the 6 miRNAs we have identified previously [12], . miR3 had the minimum read count of 235, 365, and 512 in the TDIP1, TDIP2, and TDSIP libraries, respectively. This read count was used to weed out sequences present at lower levels. Next, candidate miRNAs with overlapping sequences that do not exceed an overall length of 40 nt were compiled into a peak. The 5′ and 3′ ends with the highest read count were taken as the ends of the putative miRNA. Potential precursors (the pre-miRNA) for the putative miRNAs were generated by extracting a 100 nt sequence upstream from the 3′ end of the putative miRNA utilizing a 1 nt sliding window that shifted the sequence towards the 3′ end while maintaining a 100 nt total length. These potential pre-miRNAs were then folded using the RNAfold software to predict a hairpin structure that could be a suitable substrate for GlDcr [41], [42]. Those that failed to form a hairpin or placed the miRNA in the loop were discarded. Following this selection, 623 candidates for miRNA were identified in TDIP1, 634 in TDIP2 and 663 in TDSIP. Identical sequences, however, were found in different contigs due to overlapping sequences in GiardiaDB. Removal of duplicate sequences further reduced the number of putative miRNAs to 491, 455, and 548 for TDIP1, TDIP2, and TDSIP libraries, respectively (detailed sequence data available upon request). When the miRNA candidates from TDIP1 and TDIP2 were compared and those with the same 5′ end were chosen, the number was reduced to 208. When these sequences were compared with those from TDSIP, a pool of 105 miRNAs was identified, which included the 6 that have been already identified in our previous studies [12], [13], [14], [15], [16] (Table S1). This pool may represent the minimal number of miRNAs in *Giardia* [43], [44].

**Figure 2:**
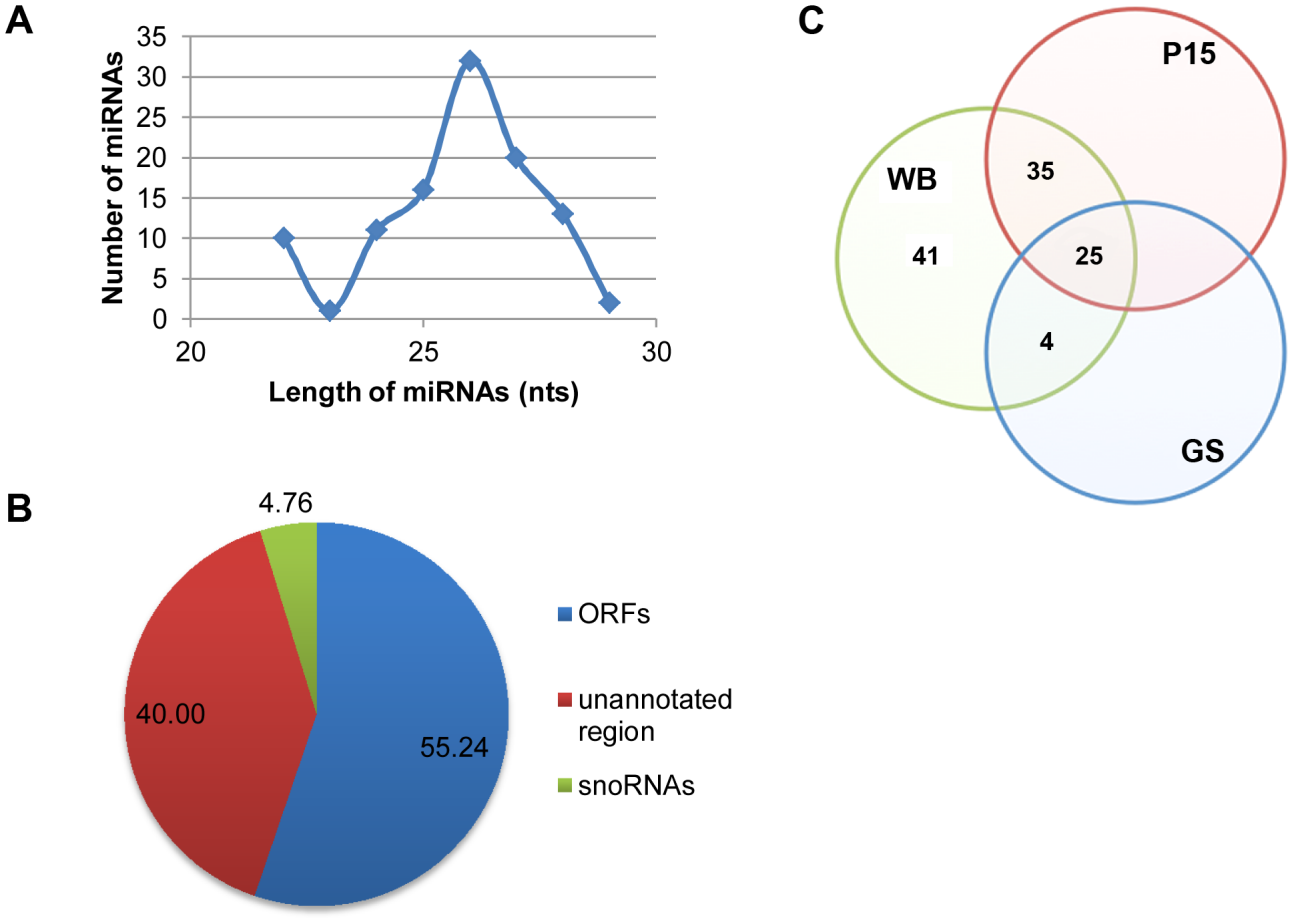
Analysis of potential miRNAs. **A**) Size distribution of the 105 miRNAs shows a range from 22–29 nts with a peak at 26 nts. **B**) Origins of the 105 miRNAs show that the majority is derived from ORFs and unannotated regions. **C**) A Venn diagram showing the conservation of potential miRNAs among the three sequenced isolates of *Giardia*. A BLAST search resulted in 41 miRNAs only in WB, 4 miRNAs conserved between WB and GS whereas 35 shared between WB and P15. A total of 25 miRNAs are conserved among all three isolates. See also Table S1.

An analysis of the sizes of the 105 putative miRNAs indicated a primary range of 24 to 28 nts with a peak population at 26 nts (Figure 2A). The 5 previously characterized miRNAs derived from snoRNAs turned out to be the only snoRNA-derived miRNAs found in the pool, constituting only 4.8% of the total (Figure 2B). There are 55.2% of the putative miRNAs derived from ORFs and 40.0% from the unannotated genomic region (Figure 2B). The results from a detailed analysis of the ORFs, presented in Table S1, suggest that many of them could be RNAs not coding for protein. Since Drosha/Pasha is known to be absent from *Giardia* [16], these likely non-coding RNAs may be first processed by GlDcr to generate the pre-miRNAs [14], and then digested by GlDcr again to produce the miRNAs [14].

In order to verify if the putative miRNAs thus identified are *bona fide* miRNAs, we used RT-qPCR to search for the previously well-identified miRNAs; miR2, miR4 [14], miR5 [12], miR6 and miR10 [13] in the ∼26 nt small RNA band co-immunoprecipitated with HA-GlAgo. Every one of these miRNAs was positively identified in the small RNA band, suggesting that most, if not all, of the miRNAs are associated with the immunoprecipitated ∼26 nt small RNA band.

Two isolates of *Giardia* other than the WB isolate, GS and P15, have also their genomes sequenced and the data incorporated into GiardiaDB. Phylogenetic studies showed that WB is more closely related to P15 than GS [45], [46]. Genomic analysis showed that average protein identity is 90% between P15 and WB but only 81% between P15 and GS [46]. We performed a BLAST search to identify putative miRNAs in GS and P15 with identical sequences to the 105 miRNAs in WB, allowing but two mismatches. The results presented in Figure 2C indicate that 25 miRNAs are conserved among all 3 isolates, whereas 41 are specific to the WB isolate. Four miRNAs are conserved between WB and GS while 35 are conserved between WB and P15 (Figure 2C). This distribution of miRNAs agrees with the results from previous phylogenetic and genetic analysis, and suggests significant diversity in the miRNA population among the three isolates.

### Identification of potential miRNA target sites among the *vsp* genes

The miRanda program was used to analyze potential target sites for the 105 miRNAs among the newly identified 73 *vsp* genes in *Giardia* [7], [47]. The miRanda analysis was first performed within a defined 3′-region of the gene (100 nts upstream and 50 nts downstream of the stop codon). It was then used to analyze the full length ORFs plus 50 nts upstream and 50 nts downstream to cover the potential 5′-UTRs and 3′-UTRs [35], [36]. The outcome indicated that multiple miRNA targeting sites are present in the 3′ region of many *vsp* genes (data not shown). Much higher numbers of miRNA target sites were identified in the full-length *vsp* ORFs with no target site found in the 5′-UTRs (data not shown). A total of 81 miRNAs were found to have potential target sites in the 3′-region while 102 of the miRNAs had potential target sites in the entire ORFs of the 73*vsp* genes. Only 3 out of the entire 105 miRNAs, miR59, miR67 and miR78, do not target any *vsp* genes. This apparent abundance of putative miRNA target sites among the *vsp* genes provided an opportunity to test whether the newly identified miRNAs exert repression on corresponding VSP expression in *Giardia*. An even more important question addresses whether interactions between the miRNAs and their target sites confined to the defined 3′-region of the VSP mRNA are enough to totally repress its expression or if the target sites upstream from this region are also involved.

### Regulation of VSP expression by targeting miRNA target sites confined to the 3′-region of mRNAs

**Figure 3:**
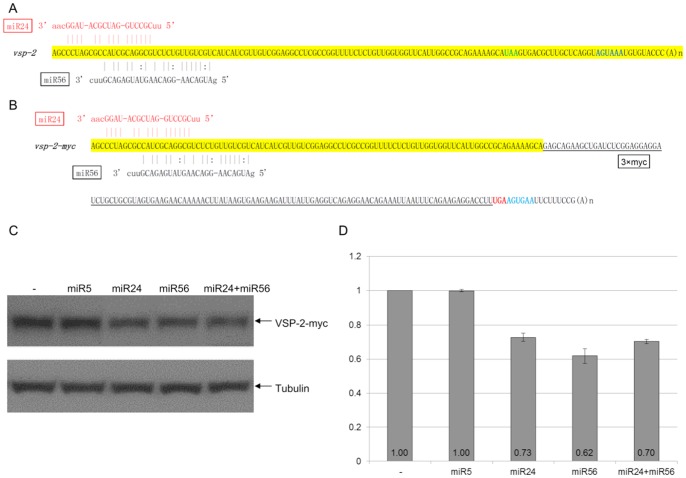
miRNA-mediated regulation of VSP-2 expression. **A**) Schematic diagram of the *vsp-2* mRNA 3′ region (100 nts upstream of the stop codon and 3′ UTR) with the binding sites for miR24 and miR56. miRNAs with fully complementary seed sequence binding sites are shown in red while miRNAs with G:U wobble pairs in the seed sequence are in black. The stop codon is shown in green and the polyadenylation signal is in blue. **B**) Schematic diagram of the *vsp-2-myc* mRNA detailing incorporation of the 3xmyc tag and elimination of the native 3′ UTR. Coloring is the same as in A). **C**) VSP-2-myc expression in *Giardia* cells transfected with the indicated miRNAs was analyzed with Western blot. Tubulin was used as a loading control. **D**) Densitometry analysis of three independent Western blot experiments is shown as a histograph with the error bars representing the standard deviation.

In previous studies, we found that VSPs tagged at the C-terminal with 3xmyc can translocate to the cell membrane similar to native VSPs [7]. An N-terminal 3xmyc tag, however, inhibited VSPs localization to the cell surface [7]. Here we choose two VSPs; VSP-2 and VSP-76 for further analysis, because they carry relatively low numbers of potential target sites at their 3′-ends and are thus easier for further analysis. Both VSPs were 3xmyc-tagged at the C-terminus (VSP-2-myc and VSP-76-myc) and episomally expressed in *Giardia* trophozoites. Both tagged VSPs could be detected on the cell membrane surface in an immunofluorescence assay (Method S1 and Figures S1A & S1B). The full-length *vsp-2* cDNA and *vsp-2-myc* cDNA were synthesized by RT-PCR and sequenced (Figures 3A & 3B). There is no putative target site for the 105 miRNAs within the short 3′-UTR of *vsp-2* mRNA (Figure 3A) or the *3xmyc* ORF and the artificial 15 nt 3′-UTR in *vsp-2-myc* mRNA (Figure 3B). The two mRNAs thus have an identical pattern of miRNA targeting sites. There are two overlapping potential target sites for miR24 and miR56 within the confinement of the 100 nts limit for the 3′-end of the *vsp-2* ORF (Figure 3A). The addition of the 126 nt *3xmyc* tag in the *vsp-2-myc* construct, however, increases the length of the *vsp* mRNA resulting in a shift of the miRNA target sites further upstream of the stop codon. Thus, by using the fusion protein, we tested target sites already beyond the original artificial limit of 100 nts upstream from the stop codon and well into the coding region.

To test the function of the newly identified miRNAs, 1 µg of synthetic miR24 and miR56 were each transfected into *Giardia* trophozoites expressing VSP-2-myc and were found to repress expression to 73% and 62% of the control, respectively (Figures 3C & 3D). When miR5, which has no target site in the *vsp-2* mRNA, was tested under the same experimental conditions, it showed no detectable effect on VSP-2-myc expression (Figures 3C & 3D), thus indicating the specificity of miRNA action. When a combination of miR24 and miR56 was introduced at 0.5 µg each, for a total of 1 µg, the expression of VSP-2-myc was repressed to 70%, indicating no additive effect, which could be attributed to the overlapping locations of the two target sites (Figures 3C & 3D). More importantly, the results also indicate that miRNA target sites located upstream from the artificially defined 100 nt limited 3′-region can still function well in interacting with the corresponding miRNAs.

**Figure 4:**
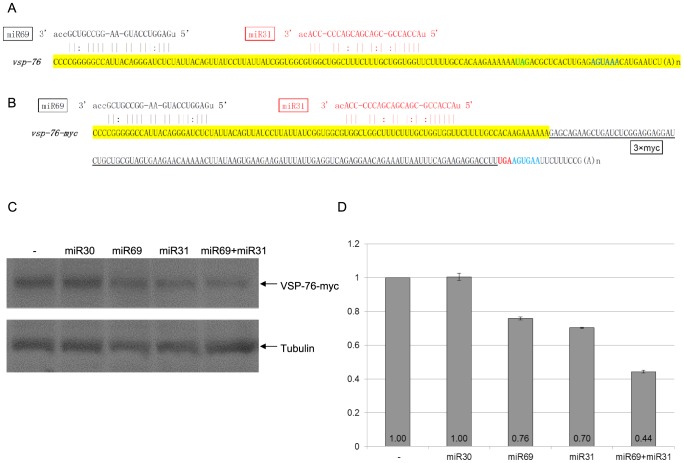
miRNA-mediated regulation of VSP-76 expression. **A**) Schematic diagram of the *vsp-2* mRNA 3′ region (100 nts upstream of the stop codon and the 3′ UTR) with the binding sites for miR24 and miR56. Coloring is as in Figure 3A. **B**) Schematic diagram of the *vsp-76-myc* mRNA detailing incorporation of the 3xmyc tag and elimination of the native 3′ UTR. **C**) *Giardia* cells expressing *vsp-76-myc* transfected with the indicated miRNAs was analyzed with Western blot. Tubulin was used as a loading control. **D**) Densitometry analysis of three independent Western blot experiments is shown as a histograph with the error bars representing the standard deviation. Expression of VSP-76-Myc is repressed in the presence of miR69 or miR31 to 76% and 70% of the control, respectively. The presence of both miRNAs represses VSP-76-Myc expression down to 44%, indicating cooperative miRNA action. miR30, which does not target *vsp-76*, does not affect VSP-76-myc expression.

The 3′-UTR of *vsp-76* mRNA also lacks any miRNA targeting site, but it consists of two separated target sites for miR69 and miR31 in the confined 3′-100 nt ORF region (Figures 4A & 4B). miR30, which does not have a target site in the vsp-76 mRNA, demonstrated no effect on VSP-76-myc expression and was used as a negative control (Figures 4C & 4D). miR69 and miR31 repressed the expression of VSP-76-myc to 76% and 70%, respectively (Figures 4C & 4D). In combination, they repressed the expression further down to 44%, thus indicating the additivity of miRNA repression when the target sites locate separately from each other (Figures 4C & 4D). But the combined repression still fails to completely knock down VSP-76-myc expression, indicating once again that interactions between target sites and miRNAs confined within the artificially defined 3′-region of the ORF may be inadequate for total repression.

### Regulation of VSP expression by targeting miRNA target sites in the entire ORF of mRNA

**Figure 5:**
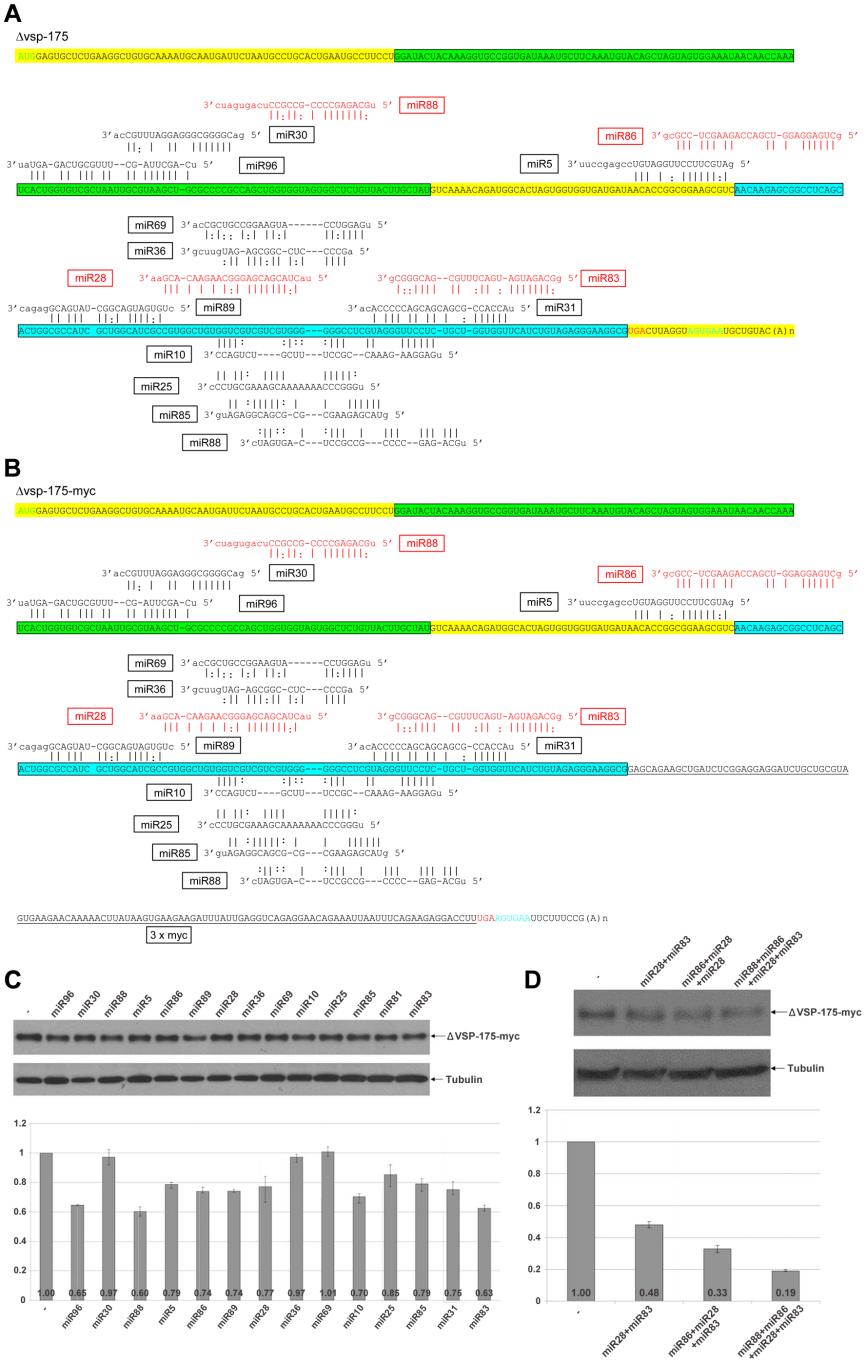
miRNA-mediated regulation of ΔVSP-175 expression. **A**) Schematic diagram of *Δvsp-175* mRNA with all 15 potential miRNA target sites indicated. miRNAs with fully complementary seed sequence binding sites are shown in red while miRNAs with G:U wobble pairs in the seed sequence are in black. Motif 1 in the VSP is highlighted in green while motif 2 in blue. The start codon is shown in green, stop codon in red and the polyadenylation signal in blue. **B**) Schematic diagram of *Δvsp-175-myc* with miRNA target sites. Coloring is the same as in A) with the 3xmyc tag underlined and the 3′ UTR in *Δvsp-175* mRNA modified. **C**) ΔVSP-175-myc expression in *Giardia* cells expressing Δ*vsp-175-myc* transfected with the indicated miRNAs was analyzed by Western blot. Tubulin was used as a loading control. Densitometry analysis of three independent Western blot experiments is shown as a histograph with error bars representing the standard deviation. **D**) The ability of miRNAs with non-overlapping target sites to cooperatively repress the expression of ΔVSP-175-myc was analyzed with Western blot analysis.

In order to learn if miRNA target sites further upstream from the confined 3′-region are required to interact with miRNAs in order to reach a higher degree of repression, we selected two relatively short *vsp* mRNAs for further analysis. One encodes a truncated version of VSP-175 (VSPA6-S) (Figure 5A). This truncated VSP (ΔVSP-175) has the N-terminal 432 amino acid residues eliminated, leaving only 20 amino acid residues upstream from motif 1 and motif 2, which are well conserved among VSPs [7]. When 3xmyc was tagged to the C-terminus and the gene (*Δvps-175-myc*) expressed episomally in transfected *Giardia* trophozoites, ΔVSP-175-myc was found localized to the cell membrane surface (Figure S1C). The cDNAs encoding ΔVSP-175 and ΔVSP-175-myc were synthesized by RT-PCR and sequenced. The results, presented in Figures 5A and 5B, indicate the absence of any miRNA target site in the 3′-UTR of *Δvsp*-175 mRNA. It and the *Δvsp-175-myc* mRNA thus consist of an identical pattern of 15 miRNA target sites throughout their respective ORFs (Figures 5A & 5B). Of the 15 target sites, two of the target sites, located far apart in the ORF, recognize the same miRNA, miR88 (Figures 5A & 5B). There are thus only 14 miRNAs acting on the two mRNAs. The 14 miRNAs were chemically synthesized and each introduced into *Giardia* trophozoites (1 µg) and assayed for repression of ΔVSP-175-myc expression. The outcome, presented in Figure 5C, shows that 11 out of the 14 miRNAs repress the expression of ΔVSP-175-myc down to a range of 60–85% when compared to the control. miR88 has the most effective repression down to 60%, which could be attributed to the presence of double non-overlapping target sites. The target site for miR96 is localized near the 5′ end of the ORF (Figure 5B). This miRNA exerts an expression repression down to 65% of the control, indicating that target sites near the 5′ end of mRNAs are also functional.

**Figure 6:**
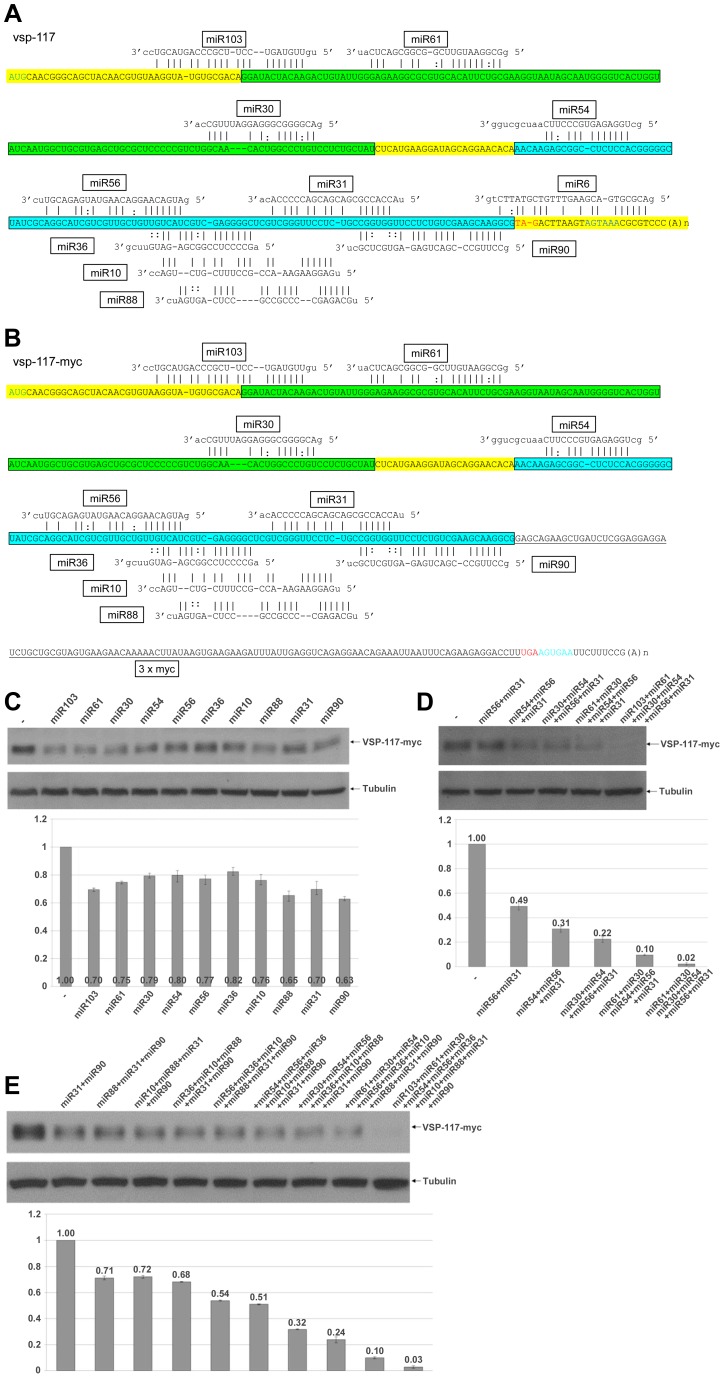
miRNA-mediated regulation of VSP-117 expression. **A**) Schematic diagram of the *vsp-117* mRNA with all 11 potential miRNA target sites. Coloring is as described in Figure 6A. **B**) Schematic diagram of the *vsp-117-myc* mRNA with 10 potential miRNA target sites. Addition of the 3xmyc tag eliminates the miR6 target site shared between the ORF and 3′-UTR. Coloring is as described above. **C**) Expression of *vsp-117-myc* transfected with the indicated miRNAs was analyzed by Western blot. Tubulin was used as a loading control. Densitometry analysis of three independent Western blot experiments is shown as a histograph with the error bars representing the standard deviation. **D**) The extent to which miRNA with non-overlapping target sites could repress express of VSP-117-myc was tested. The results indicate that a combined action of 6 miRNAs is sufficient to nearly totally eliminate the mRNA expression. **E**) Co-transfection of miRNA combinations with overlapping target sites repressed VSP-117-myc expression without cooperative effect. A combination of all 10 miRNAs progressively decreases the expression of VSP-117-myc to near zero.

The 3 miRNAs, miR30, miR36 and miR69, that failed to show any appreciable effect on ΔVSP-175-myc expression (Figure 5C), however, could effectively repress expression of VSP-117-myc (see Figure 6C for miR30 and miR36) or VSP-76-myc (Figure 4D for miR69). Their failure in repressing ΔVSP-175-myc expression could be attributed to different locations of the target site in different mRNAs (see Discussion).

When combined, effects of the miRNAs on non-overlapping target sites were measured. miR28 and miR83 (0.5 µg of each) repressed ΔVSP-175-myc expression down to 48%, miR86+miR28+miR83 (0.33 µg of each) down to 33% and miR88+miR86+miR28+miR83 (0.25 µg of each) down to 19% (Figure 5D). The additivity of inhibitory effects among the miRNAs is thus applicable to the non-overlapping target sites throughout the entire ORF of the mRNA (Figure 5A). More than 80% repression of expression was achieved by a combination of the four miRNAs.

In order to verify if the miRNA repressive effect could be reversed by its corresponding 2′-O-methyl antisense oligo (ASO), the ASOs of miR5 and miR10 were each co-transfected with the corresponding miRNA at an equal concentration. The results showed a total reversal of the inhibitory effects of both miR5 [12] and miR10 [13], indicating the specificity of miRNA action.

We then sequenced another cDNA encoding VSP-117 (GL50803_97820), which consists of 309 bp and 11 putative miRNA target sites in the entire ORF and across the 3′-UTR (Figure 6A). When it is compared with the cDNA sequence of *vsp-117-myc*, a miR6 target site located across the stop codon in *vsp-117* mRNA was elimated due to the 3xmyc tagging (Figure 6B). The miR6 target site has been found located across the stop codons of multiple *vsp* mRNAs. miR6 and its target site in the mRNAs of VSP-1267 and VSP-213, tagged at the N-terminus with 3xmyc, have been analyzed in our previous studies and shown to repress expression by 30% in each case [13]. VSP-117-myc was localized to the cell membrane similar to the other tagged VSPs (Figure S1D).

An ASO of miR6 was also co-transfected with the miRNA. The result indicated a total abolition of the repressive effect of miR6 [13], verifying the specificity of miR6 action.

We tested the ability of the10 miRNAs other than miR6 to independently repress the expression of VSP-117-myc as described above. The results indicate that every miRNA exerts a partial repression of expression ranging from 63 to 82% of the control (Figure 6C). We then tested combinations of miRNAs with non-overlapping target sites. The data show that when increasing numbers of miRNAs are included in the combination, a higher extent of repression is observed (Figure 6D). When six miRNAs with non-overlapping target sites, miR103+miR61+miR30+miR54+miR56+miR31, were combined at 0.17 µg each, the expression of VSP-117-myc was repressed down to 2% of the control level (Figure 6D). A near total repression was thus achieved even though not all the available target sites were occupied by miRNAs. The 6 miRNAs with non-overlapping target sites are sufficient to nearly completely eliminate mRNA expression. The most intriguing finding is perhaps that near total repression was achieved even when the miR6 target site at the 3′-UTR was eliminated and miR6 was not involved in targeting the mRNA. When additional miRNAs with target sites overlapping the target sites of the initial 6 miRNAs were added, they did not further enhance the level of repression (Figure 6E). Thus, when all 10 miRNAs that target *vsp-117-myc* were combined at 0.1 µg each, expression of VSP-117-myc was repressed to 3%. This is not significantly different from the extent of repression when only the 6 miRNAs with non-overlapping target sites were included. This outcome suggests that multiple miRNAs with overlapping target sites could competitively regulate expression of *vsp* mRNAs. The redundant miRNAs and miRNA target sites could function to increase the rate of target site occupancy.

We thus conclude that essentially all the target sites in the ORF of *vsp* mRNAs may be functional. They do not, however, need to be fully occupied in order to achieve total repression of the mRNA expression. Occupation of some of the non-overlapping target sites throughout the ORF should be sufficient to eliminate mRNA expression. The presence of additional target sites at overlapping positions, however, may allow different combinations of miRNAs to effectively repress expression of the *vsp* mRNA. This could be a means of maintaining VSP expression in a repressed state while the levels of miRNAs may fluctuate. This is also the first time, as far as we are aware, that miRNA target sites throughout the entire ORF are found to be functional and essential for full repression of mRNA expression.

## Discussion

We have successfully constructed 3 libraries of the GlAgo associated small RNAs from *Giardia* WB trophozoites for next generation sequencing (NGS). The numbers of raw reads, preprocessed reads, aligned reads and unique reads derived from each of the 3 independent libraries appear highly similar (see Table 1). This similarity suggests high quality and reproducibility of data among the 3 libraries and a high degree of reliability in the number and identity of reads derived from them. The 105 miRNAs resulted from the final analysis could be thus not far off from the actual number of miRNAs in the WB isolate of *Giardia*.

About two dozens of the newly identified miRNAs have since undergone *in vivo* tests for their potential activities in repressing the expression of *vsp* mRNAs carrying the corresponding target sites. All of them demonstrated the ability to repress their target mRNAs, indicating their authenticity and suggesting that all the 105 miRNAs could be *bona fide* miRNAs in *Giardia*. The extent of *vsp* mRNA repression by these miRNAs is usually in the range of 20 to 40%, similar to those we have observed previously with the 5 snoRNA-derived miRNAs, suggesting that the origins of miRNAs do not significantly affect the potencies of miRNAs.

The data from analyzing miRNA actions on *Δvsp-175-myc* and *vsp-117-myc* expressions also indicate that a miRNA acting on a target site close to the 5′-end of ORF is just as effective as a target site close to the 3′-end (Figures 5C and 6C). This is in sharp contrast to the observations made in higher eukaryotes, which have the majority of miRNA target sites located in the 3′-UTR [20], [21], a few in the 3′-end of coding region [22], [25], [26], [28], [29], [30],,but none in the 5′-end of ORFs. Thus, even though up to 50% of potential miRNA target sites have been identified in the ORFs by HITS-CLIP analysis, they have not yet been extensively tested and assumed to be largely nonfunctional [32], [33]. This assumed lack of function of the miRNA target sites in the 5′ portion of ORF was attributed to active translation, which impedes miRNA-programmed miRISC association with target mRNAs [31]. Such an explanation cannot be, however, applied to the active miRNA function on the target sites throughout the ORF of *vsp* mRNAs in *Giardia*. The mechanism of translational initiation in *Giardia* does differ from that in the higher eukaryotes in certain aspects. There is apparently no mechanism of ribosome scanning required for searching of the initiation codon in order to start translation in *Giardia* [48]. The highest translational efficiency is achieved when AUG is positioned immediately in front of the ORF [48], which is also reflected by the relatively short 5′-UTRs among the mRNAs in *Giardia* [35], [36]. Whether this interesting feature in translational initiation in *Giardia* may contribute to the apparent importance of miRNA and target site interactions at the 5′-end of ORFs remains to be seen. Overall, a more thorough analysis of the miRNA target sites in the 5′-region of ORFs appears to be in order among higher eukaryotes at this time.

The 3′-UTRs in *Giardia* mRNAs have an average length of 30 nts [35], [36]. Thus, in most cases, they are apparently barely long enough to accommodate a single miRNA target site. To date, we have been unable to identify in *Giardia* a miRNA target site located completely within the 3′ UTR. Instead, 3′ UTR target sites usually straddle the stop codon with the miRNA seed sequence binding in the 3′ UTR and the remaining miRNA sequence binding to the 3′ end of ORF. A recent publication extended the median size of *Giardia* 3′ UTRs to 80 nts [37], which may indicate the potential presence of miRNA target sites in the 3′-UTRs of some mRNAs. In view of the partial repression exerted by the interaction between a single miRNA and a single target site and the lack of additivity between miRNA actions on overlapping target sites, it is not possible for *Giardia* to rely only on miRNA action in the 3′-UTR of mRNA alone for effective control of post-transcriptional gene expression. In fact, among the 4 *vsp* mRNAs we have analyzed in the present study, 3 do not have a target site in their 3′-UTRs at all. This may make it necessary for the target sites in the ORF of *vsp* mRNAs to be functional in order to achieve total repression of its expression in *Giardia*. There have been some recent examples of miRNA actions on target sites in the ORFs of mammalian mRNAs. They include the action of miR-183 on *βTrCP1* mRNA in colorectal cancer cells [23], the action of miR-143 on mouse pleiotrophin mRNA [27] and the action of miR-34a on MDM4 mRNA in colon carcinoma cells [24]. Though the data are still too preliminary to reveal any potential significance in miRNA target sites localized to the upstream portion of ORF, further studies may throw more light on the subject.

We have also noted in the current as well as previous studies that the locations of some miRNA target sites are highly conserved among different *vsp* mRNAs. For example, the miR10 target site in all the *vsp* mRNAs is typically located at positions −62 to −32 upstream from the stop codon, whereas the miR6 target site is usually located across the stop codon [13]. Similarly, the majority of miR69 target sites are located at positions −100 to −75. When it is not located at this position in *Δvsp-*175-*myc* mRNA, it was found not functional (see Figure 5). The location of miR36 target site in *Δvsp-*175-*myc* and *vsp-117* is shifted by only one nucleotide. It was found functional in *vsp-117* but not in *Δvsp-*175-*myc*. Finally, the miR30 target site is located at different positions upstream from the 100 nt 3′-end region in *Δvsp-*175-*myc* and *vsp-117* and showed functional differences (see Figures 5 & 6). These differences in target site functionality could be attributed to differences in mRNA secondary structure and distinctions in thermodynamic stability of miRNA binding.

Our current observations have provided a tentative explanation for how expression of *vsp* mRNAs is regulated in *Giardia* trophozoites. All 73 *vsp* mRNAs carrying multiple target sites in their ORFs for miRNAs are likely present in the trophozoites [7]. A variety of combinations of miRNAs from among the 102 miRNAs could effectively repress the expression of each of the *vsp* mRNAs. Not all the target sites in an ORF need to be saturated with miRNAs, however, and a significant extent of miRNA redundancy could maintain most of the *vsps* in a silent state (see Figure 6). The example presented in Figure 6 indicates that only 6 miRNAs with non-overlapping target sites in the ORF are sufficient to bring about near total repression of mRNA expression. This repression does not even require the interaction between miR6 and its target site located partly in the 3′-UTR. Thus, the mechanism of miRNA-mediated translational repression in *Giardia* targets apparently the entire coding region of the mRNA. This could require mechanisms of miRISC action not present or not yet been explored in higher eukaryotes.

In all, our current study has determined the pool of miRNAs in *Giardia*, the tentative mechanism of miRNA repression of VSP expression in *Giardia*, and, most of all, a likely first indication that miRNAs can act on target sites throughout the entire ORF of a mRNA.

## Materials and Methods

### NGS library construction

The NGS libraries of GlAgo associated small RNAs were constructed using a modified version of the Mello Lab protocol (www.lsi.umich.edu/files/SmallRNACloning.pdf). Briefly, the GlAgo associated small RNAs were isolated from *Giardia* WB trophozoites as previously described and resuspended in 10 µl RNase-free diH_2_O [14]. For the addition of a sonication step to the procedure, the cells were resuspend in RIPA buffer as described previously [14] and sonicated twice with five 1 second bursts at a 20% duty cycle. The purified small RNAs were ligated at the 3′-end to 5–10 µM gel purified miRNA Cloning Linker-1 (IDT) using T4 RNA ligase 2 and truncated with K227Q (NEB) by incubating at 25°C for 1 hr, 15°C for 2 hrs and followed by a final 4°C incubation overnight. The reaction mixture was analyzed in 15% polyacrylamide/8 M Urea gel, and the band of ligated product was excised. The gel slice was crushed, resuspended in 1× TE buffer along with 1 U/µl SUPERase-In (Life technologies), and incubated with shaking at 37°C overnight. The reaction mixture was then transferred to a Spin-X cellulose acetate filter centrifuge tubes to remove the gel fragments, RNA was precipitated using ethanol and resuspended in 5 µl RNase-free diH_2_O. The small RNA with 3′-adaptor was then ligated to the 5′ adaptor (TCTACrArGrUrCrCrGrArCrGrArUrC; r IDT) by incubating with 1 µl T4 RNA ligase 1 at 37°C for 15 min. This step was repeated and the reaction was incubated at 15°C for 2 hrs followed by a final incubation at 4°C overnight. The product was analyzed and extracted as described above and resuspended in 10 µl RNase-free diH_2_O. The resulting 3′ and 5′ adaptor linked small RNAs (2.5 µl) were reverse transcribed using an RT DNA oligo (ATT GAT GGT GCC TAC AG) with Superscript III (Life Technologies) as per the manufacturer recommended protocol. Of the 20 µl cDNA reaction, 5 µl was PCR amplified with the RT DNA oligo and DNA oligo (GTT CTA CAG TCC GAC GAT C) primers for 10 cycles (94°C, 1 min; 94°C, 20 sec; 50°C, 20 sec; 68°C, 20 sec) using Platinum Taq (Life Technologies). Following the 10 cycles, Illumina 5′ (AAT GAT ACG GCG ACC ACC GAC AGG TTC AGA GTT CTA CAG TCC GAC GAT C) and Illumina 3′ (CAA GCA GAA GAC GGC ATA CGA ATT GAT GGT GCC TAC AG) primers were added to the reaction and incubated for an additional 9 cycles to incorporate the Illumina adapter sequences. The PCR reaction mixture was analyzed in native 10% polyacrylamide gel, and the amplified band of cDNA library was purified. The library was then analyzed using the Agilent Bioanalyzer with the Agilent High Sensitivity DNA kit. The library concentration was adjusted and sequenced using the Illumina HiSeq 2500.

### Reads trimming and filtering

Over 190 million raw reads were obtained for each library. They were filtered to retain only the high quality reads. First, the low quality ends (Q = 2) in the 5′ half of the read were discarded. The reads with an average quality less than Q = 20 were then discarded. The 3′ adapter sequence was removed. Reads shorter than the length of a typical miRNA (<20 nt) were then discarded. This resulted in approximately 5% of reads discarded from each library.

### Aligning reads to the *Giardia* scaffolds

Some of the trimmed and filtered reads contained polyA tails of variable lengths, whereas some of the others had the 3′-end of tRNA ‘CCA’. A zero-mismatch alignment using Bowtie [38] against the *G. lamblia* scaffolds (obtained from GiardiaDBv2.5) [39] was applied:

1. The reads were aligned without mismatch to the scaffolds. Non-aligning reads that did not end with an ‘A’ nucleotide at the 3′ end were discarded.
2. For the reads ending with an ‘A’ from the previous step, the ‘A’ was removed. Any reads shorter than 20 nt were discarded. The remaining reads were then aligned without mismatch to the scaffolds. Non-aligning reads were again separated into two files based on whether the 3′ nucleotides ended with an ‘A’ or a ‘CC’. Reads not ending in these nucleotides were discarded.
3. For the reads ending with a ‘CC’ from the previous step, the ‘CC’ nucleotides were removed and the reads were aligned without mismatch to only the tRNA sequences.
4. Steps two and three were repeated until there were no reads remaining.

### Identification of putative miRNAs

Putative miRNAs candidates in the 3 libraries were selected by initially determining the minimum read counts for known miRNAs. Only sequences with similar or higher read counts were retained to eliminate those from random degradations present at lower levels. When all the reads are combined, peaks with overlapping reads that do not exceed 40 nt were identified. Exceptions such as the ORF for miR4 which contains a significant number reads throughout the RNA (see below), however, do exist in the library. These regions were analyzed similarly when individual peaks could be identified. The 5′ and 3′ ends were determined by taking the read with the highest read count in the isolated peak. Potential precursors for each putative miRNA were generated by extracting a 100 nt sequence with the potential miRNA at the 3′ end. Additional potential precursors were generated by utilizing a 1 nt sliding window that shifted the sequence towards the 3′ end while maintaining a 100 nt total length. Sequences were generated until the potential miRNA was located at the 5′ end. These potential precursors were then folded using RNAfold to predict hairpin structures [41]. Potential miRNAs that did not form hairpin structures that could be GlDcr substrates were discarded. Hairpin structures where the potential miRNA is located in the loop were also discarded. An exact match of miRNA sequences among the three libraries were chosen to be the *bona fide* miRNAs.

### Target prediction

Targets of miRNA candidates were predicted with miRanda [47] on either the 3′ region of each ORF (100 nts upstream and 50 nts downstream of the stop codon), or the full length ORF plus 50 nts upstream and 50 nts downstream of the coding region.

### Cloning of 3xmyc-tagged *vsp* genes

The entire coding region of each chosen *vsp* gene was PCR amplified from *Giardia* genomic DNA using specific primers. The product was cloned into pGEM-T Easy (Promega), sequenced and sub-cloned into the pNlop4 vector used in our previous studies [7]. The pNlop4 vector was modified to introduce the *vsp* gene and the 3xmyc epitope upstream from the TAA stop codon.

### Cell culture, transfection and selection

*Giardia* (WB clone C6, ATCC50803) trophozoites were grown anaerobically in plastic culture tubes at 37°C in the modified TYI-S-33 medium supplemented with antibiotics as described [49]. Transfections of *Giardia* trophozoites were carried out using electroporation as previously described [13]. Briefly, cells were harvested and washed twice in phosphate buffered saline (PBS) and once in electroporation buffer (Cytomix buffer: 10 mM K_2_HPO_4_–KH_2_PO_4_ pH 7.6, 25 mM HEPES free acid, 120 mM KCl, 0.15 mM CaCl_2_, 2 mM EGTA, 5 mM MgCl_2_, 2 mM ATP, 4 mM glutathione). A 400 µl cell suspension (containing 10^7^ cells) was transferred to a 0.2-cm-gap electroporation cuvette and a sample of 50 µg plasmid DNA or yeast tRNA (125 µg) and synthetic 5′-phosphate-miRNA (1 µg) was added to the cell suspension. The cells were immediately subjected to electroporation using a Bio-Rad Gene Pulser Xcell (Voltage: 450 V, Capacitance: 500 mF, Resistance: ∞). The electroporated cells were incubated on ice for 10 min, added to pre-warmed culture medium, and incubated at 37°C. For selection, 200 µg/ml G418 was added to the medium 16 hrs after transfection. The selected cells were incubated with 5 µg/ml of tetracycline at 37°C for 16 hrs to induce expression of the cloned gene.

To ensure a consistent transfection efficiency, a RLuc expressing plasmid was co-transfected with or without the miRNAs in repeated control experiments. The results were always highly reproducible [12], [13], [14], [15], [16]. The miRNAs used for transfection were in the saturating level, which gave also highly reproducible outcomes.

### Western blot

*Giardia* trophozoites containing episomally expressed vsp genes were transfected with the appropriate miRNAs and incubated for 16–20 hrs at 37°C in pre-warmed medium containing tetracycline (5 µg/ml). The cells were cooled on ice, pelleted, washed once with cold PBS and resuspended in lysis buffer (50 mM Tris-HCl (pH 7.4), 150 mM NaCl, 0.5% sodium deoxycholate, 1% NP-40, 1×Halt protease inhibitor cocktail (Thermo Scientific)) and incubated at 4°C for 30 min. The cell lysate was cleared by centrifugation at 16,000 g at 4°C for 20 min. For SDS-PAGE separation, 25 µg of protein from each sample was used. The protein was transferred to a PVDF membrane (Bio-Rad) for detection with the anti-c-myc-HRP antibody (Invitrogen). It was subsequently stripped using Restore Western Blot Stripping Buffer (Thermo Scientific) and re-blotted with anti-Tubulin antibody (Sigma) for loading control. Protein bands were quantified using the BioRad Quantity One software package. The results were derived from statistical analysis of three independent experiments. Margins of errors in the data from these experiments were generally quite small, which support confidence in the accuracy of the data.

### Accession numbers

The pre-processed have been deposited into the *Giardia* database (GiardiaDB.org) a part of the EuPathDB project.

## Supporting Information

Figure S1 Immunofluorescent localization of C-terminal 3xmyc tagged VSPs (**A, B, C, and D**). All four 3xmyc tagged VSPs are expressed and localized to the membrane surface of *Giardia* trophozoites.
(TIF)

Method S1 Immunofluorescence assay. For detecting the C-terminal 3xmyc tagged VSP expression, indirect immuno-staining of nonpermeabilized cells were carried out as previously described (Li W, *et al*., in press). Briefly, the harvested *Giardia* cells were adhered to a poly-L-lysine coated cover slip (BD Biosciences). The cells were then fixed with 3% paraformaldehyde in NaPi (100 mM NaPi, pH 7.4) at room temperature, washed with NaPi, blocked with 0.2% gelatin in NaPi and immuno-stained using 1∶500 diluted anti-myc-FITC antibody (Invitrogen) in 0.2% gelatin in NaPi. The immuno-stained cells were examined using a Nikon TE2000E motorized inverted microscope equipped with 60×bright field and epifluorescence optics. Images were acquired with the NIS-Elements Advanced Research software (Nikon).
(DOC)

Table S1 Analysis of the 105 putative miRNAs identified in *Giardia*. The ones colored in yellow have also been identified in our previous studies.
(XLSX)

Table S2 Number of miRNA reads in each library. A tabulation of the numbers of reads of each of the 105 miRNAs in each of the three libraries; D1, D2 and SD1.
(XLSX)

## References

[1] Barwick RS, Levy DA, Craun GF, Beach MJ, Calderon RL (2000) Surveillance for waterborne-disease outbreaks–United States, 1997–1998. MMWR CDC Surveill Summ 49: 1–21. 10843502

[2] Savioli L, Smith H, Thompson A (2006) Giardia and Cryptosporidium join the ‘Neglected Diseases Initiative’. Trends Parasitol 22: 203–208. 16545611 10.1016/j.pt.2006.02.015

[3] Adam RD, Nigam A, Seshadri V, Martens CA, Farneth GA, et al (2010) The Giardia lamblia vsp gene repertoire: characteristics, genomic organization, and evolution. BMC Genomics 11: 424. 20618957 10.1186/1471-2164-11-424 PMC2996952

[4] Nash TE, Banks SM, Alling DW, Merritt JW Jr, Conrad JT (1990) Frequency of variant antigens in Giardia lamblia. Exp Parasitol 71: 415–421. 1699782 10.1016/0014-4894(90)90067-m

[5] Nash TE, Lujan HT, Mowatt MR, Conrad JT (2001) Variant-specific surface protein switching in Giardia lamblia. Infect Immun 69: 1922–1923. 11179375 10.1128/IAI.69.3.1922-1923.2001 PMC98104

[6] Deitsch KW, Lukehart SA, Stringer JR (2009) Common strategies for antigenic variation by bacterial, fungal and protozoan pathogens. Nat Rev Microbiol 7: 493–503. 19503065 10.1038/nrmicro2145 PMC3676878

[7] Li W, Saraiya AA, Wang CC (2013) Experimental Verification of the Identity of Variant-Specific Surface Proteins in Giardia lamblia Trophozoites. MBio 4: e00321–13. 23695837 10.1128/mBio.00321-13 PMC3656445

[8] Mowatt MR, Aggarwal A, Nash TE (1991) Carboxy-terminal sequence conservation among variant-specific surface proteins of Giardia lamblia. Mol Biochem Parasitol 49: 215–227. 1775165 10.1016/0166-6851(91)90065-e

[9] Nash TE, Mowatt MR (1992) Characterization of a Giardia lamblia variant-specific surface protein (VSP) gene from isolate GS/M and estimation of the VSP gene repertoire size. Mol Biochem Parasitol 51: 219–227. 1574080 10.1016/0166-6851(92)90072-r

[10] Prucca CG, Slavin I, Quiroga R, Elias EV, Rivero FD, et al (2008) Antigenic variation in Giardia lamblia is regulated by RNA interference. Nature 456: 750–754. 19079052 10.1038/nature07585

[11] Faghiri Z, Widmer G (2011) A comparison of the Giardia lamblia trophozoite and cyst transcriptome using microarrays. BMC Microbiol 11: 91. 21542940 10.1186/1471-2180-11-91 PMC3096902

[12] Li W, Saraiya AA, Wang CC (2011) Gene regulation in Giardia lambia involves a putative microRNA derived from a small nucleolar RNA. PLoS Negl Trop Dis 5: e1338. 22028939 10.1371/journal.pntd.0001338 PMC3196473

[13] Li W, Saraiya AA, Wang CC (2012) The profile of snoRNA-derived microRNAs that regulate expression of variant surface proteins in Giardia lamblia. Cell Microbiol 14: 1455–73. 22568619 10.1111/j.1462-5822.2012.01811.x PMC3422372

[14] Saraiya AA, Li W, Wang CC (2011) A microRNA derived from an apparent canonical biogenesis pathway regulates variant surface protein gene expression in Giardia lamblia. RNA 17: 2152–2164. 22033329 10.1261/rna.028118.111 PMC3222128

[15] Saraiya AA, Li W, Wang CC (2013) Transition of a microRNA from repressing to activating translation depending on the extent of base pairing with the target. PLoS One 8: e55672. 23405193 10.1371/journal.pone.0055672 PMC3565978

[16] Saraiya AA, Wang CC (2008) snoRNA, a novel precursor of microRNA in Giardia lamblia. PLoS Pathog 4: e1000224. 19043559 10.1371/journal.ppat.1000224 PMC2583053

[17] Krol J, Loedige I, Filipowicz W (2010) The widespread regulation of microRNA biogenesis, function and decay. Nat Rev Genet 11: 597–610. 20661255 10.1038/nrg2843

[18] Bazzini AA, Lee MT, Giraldez AJ (2012) Ribosome profiling shows that miR-430 reduces translation before causing mRNA decay in zebrafish. Science 336: 233–237. 22422859 10.1126/science.1215704 PMC3547538

[19] Djuranovic S, Nahvi A, Green R (2012) miRNA-mediated gene silencing by translational repression followed by mRNA deadenylation and decay. Science 336: 237–240. 22499947 10.1126/science.1215691 PMC3971879

[20] Bartel DP (2009) MicroRNAs: target recognition and regulatory functions. Cell 136: 215–233. 19167326 10.1016/j.cell.2009.01.002 PMC3794896

[21] Pasquinelli AE (2012) MicroRNAs and their targets: recognition, regulation and an emerging reciprocal relationship. Nat Rev Genet 13: 271–282. 22411466 10.1038/nrg3162

[22] Duursma AM, Kedde M, Schrier M, le Sage C, Agami R (2008) miR-148 targets human DNMT3b protein coding region. RNA 14: 872–877. 18367714 10.1261/rna.972008 PMC2327368

[23] Elcheva I, Goswami S, Noubissi FK, Spiegelman VS (2009) CRD-BP protects the coding region of betaTrCP1 mRNA from miR-183-mediated degradation. Mol Cell 35: 240–246. 19647520 10.1016/j.molcel.2009.06.007 PMC2742352

[24] Mandke P, Wyatt N, Fraser J, Bates B, Berberich SJ, et al (2012) MicroRNA-34a modulates MDM4 expression via a target site in the open reading frame. PLoS One 7: e42034. 22870278 10.1371/journal.pone.0042034 PMC3411609

[25] Shen WF, Hu YL, Uttarwar L, Passegue E, Largman C (2008) MicroRNA-126 regulates HOXA9 by binding to the homeobox. Mol Cell Biol 28: 4609–4619. 18474618 10.1128/MCB.01652-07 PMC2447122

[26] Tay YM, Tam WL, Ang YS, Gaughwin PM, Yang H, et al (2008) MicroRNA-134 modulates the differentiation of mouse embryonic stem cells, where it causes post-transcriptional attenuation of Nanog and LRH1. Stem Cells 26: 17–29. 17916804 10.1634/stemcells.2007-0295

[27] Yi C, Xie WD, Li F, Lv Q, He J, et al (2011) MiR-143 enhances adipogenic differentiation of 3T3-L1 cells through targeting the coding region of mouse pleiotrophin. FEBS Lett 585: 3303–3309. 21945314 10.1016/j.febslet.2011.09.015

[28] Jung HM, Patel RS, Phillips BL, Wang H, Cohen DM, et al (2013) Tumor suppressor miR-375 regulates MYC expression via repression of CIP2A coding sequence through multiple miRNA-mRNA interactions. Mol Biol Cell 24: 1638–1637, 1638-1648, S1631-1637. 23552692 10.1091/mbc.E12-12-0891 PMC3667718

[29] Qin W, Shi Y, Zhao B, Yao C, Jin L, et al (2010) miR-24 regulates apoptosis by targeting the open reading frame (ORF) region of FAF1 in cancer cells. PLoS One 5: e9429. 20195546 10.1371/journal.pone.0009429 PMC2828487

[30] Forman JJ, Legesse-Miller A, Coller HA (2008) A search for conserved sequences in coding regions reveals that the let-7 microRNA targets Dicer within its coding sequence. Proc Natl Acad Sci U S A 105: 14879–14884. 18812516 10.1073/pnas.0803230105 PMC2567461

[31] Gu S, Jin L, Zhang F, Sarnow P, Kay MA (2009) Biological basis for restriction of microRNA targets to the 3′ untranslated region in mammalian mRNAs. Nat Struct Mol Biol 16: 144–150. 19182800 10.1038/nsmb.1552 PMC2713750

[32] Chi SW, Zang JB, Mele A, Darnell RB (2009) Argonaute HITS-CLIP decodes microRNA-mRNA interaction maps. Nature 460: 479–486. 19536157 10.1038/nature08170 PMC2733940

[33] Zisoulis DG, Lovci MT, Wilbert ML, Hutt KR, Liang TY, et al (2010) Comprehensive discovery of endogenous Argonaute binding sites in Caenorhabditis elegans. Nat Struct Mol Biol 17: 173–179. 20062054 10.1038/nsmb.1745 PMC2834287

[34] Fang Z, Rajewsky N (2011) The impact of miRNA target sites in coding sequences and in 3′UTRs. PLoS One 6: e18067. 21445367 10.1371/journal.pone.0018067 PMC3062573

[35] Adam RD (2000) The Giardia lamblia genome. Int J Parasitol 30: 475–484. 10731570 10.1016/s0020-7519(99)00191-5

[36] Adam RD (2001) Biology of Giardia lamblia. Clin Microbiol Rev 14: 447–475. 11432808 10.1128/CMR.14.3.447-475.2001 PMC88984

[37] Franzen O, Jerlstrom-Hultqvist J, Einarsson E, Ankarklev J, Ferella M, et al (2013) Transcriptome profiling of Giardia intestinalis using strand-specific RNA-seq. PLoS Comput Biol 9: e1003000. 23555231 10.1371/journal.pcbi.1003000 PMC3610916

[38] Langmead B, Trapnell C, Pop M, Salzberg SL (2009) Ultrafast and memory-efficient alignment of short DNA sequences to the human genome. Genome Biol 10: R25. 19261174 10.1186/gb-2009-10-3-r25 PMC2690996

[39] Aurrecoechea C, Brestelli J, Brunk BP, Carlton JM, Dommer J, et al (2009) GiardiaDB and TrichDB: integrated genomic resources for the eukaryotic protist pathogens Giardia lamblia and Trichomonas vaginalis. Nucleic Acids Res 37: D526–530. 18824479 10.1093/nar/gkn631 PMC2686445

[40] Macrae IJ, Zhou K, Li F, Repic A, Brooks AN, et al (2006) Structural basis for double-stranded RNA processing by Dicer. Science 311: 195–198. 16410517 10.1126/science.1121638

[41] Hofacker IL, Fontana W, Stadler PF, Bonhoeffer LS, Tacker M, et al (1994) Fast folding and comparison of RNA secondary structures. Monatshefte für Chemie/Chemical Monthly 125: 167–188.

[42] Hofacker IL, Stadler PF (2006) Memory efficient folding algorithms for circular RNA secondary structures. Bioinformatics 22: 1172–1176. 16452114 10.1093/bioinformatics/btl023

[43] MacRae IJ, Zhou K, Doudna JA (2007) Structural determinants of RNA recognition and cleavage by Dicer. Nat Struct Mol Biol 14: 934–940. 17873886 10.1038/nsmb1293

[44] Park JE, Heo I, Tian Y, Simanshu DK, Chang H, et al (2011) Dicer recognizes the 5′ end of RNA for efficient and accurate processing. Nature 475: 201–205. 21753850 10.1038/nature10198 PMC4693635

[45] Ey PL, Mansouri M, Kulda J, Nohynkova E, Monis PT, et al (1997) Genetic analysis of Giardia from hoofed farm animals reveals artiodactyl-specific and potentially zoonotic genotypes. J Eukaryot Microbiol 44: 626–635. 9435134 10.1111/j.1550-7408.1997.tb05970.x

[46] Jerlstrom-Hultqvist J, Franzen O, Ankarklev J, Xu F, Nohynkova E, et al (2010) Genome analysis and comparative genomics of a Giardia intestinalis assemblage E isolate. BMC Genomics 11: 543. 20929575 10.1186/1471-2164-11-543 PMC3091692

[47] John B, Enright AJ, Aravin A, Tuschl T, Sander C, et al (2004) Human MicroRNA targets. PLoS Biol 2: e363. 15502875 10.1371/journal.pbio.0020363 PMC521178

[48] Li L, Wang CC (2004) Capped mRNA with a single nucleotide leader is optimally translated in a primitive eukaryote, Giardia lamblia. J Biol Chem 279: 14656–14664. 14722094 10.1074/jbc.M309879200

[49] Keister DB (1983) Axenic culture of Giardia lamblia in TYI-S-33 medium supplemented with bile. Trans R Soc Trop Med Hyg 77: 487–488. 6636276 10.1016/0035-9203(83)90120-7

