# Supplementary figures and images for: The microRNAs in an Ancient Protist Repress the Variant-Specific Surface Protein Expression by Targeting the Entire Coding Sequence
Source: PLoS Pathog. 2014 Feb 27;10(2):e1003791. doi: 10.1371/journal.ppat.1003791 (PMC3937270; doi:10.1371/journal.ppat.1003791)

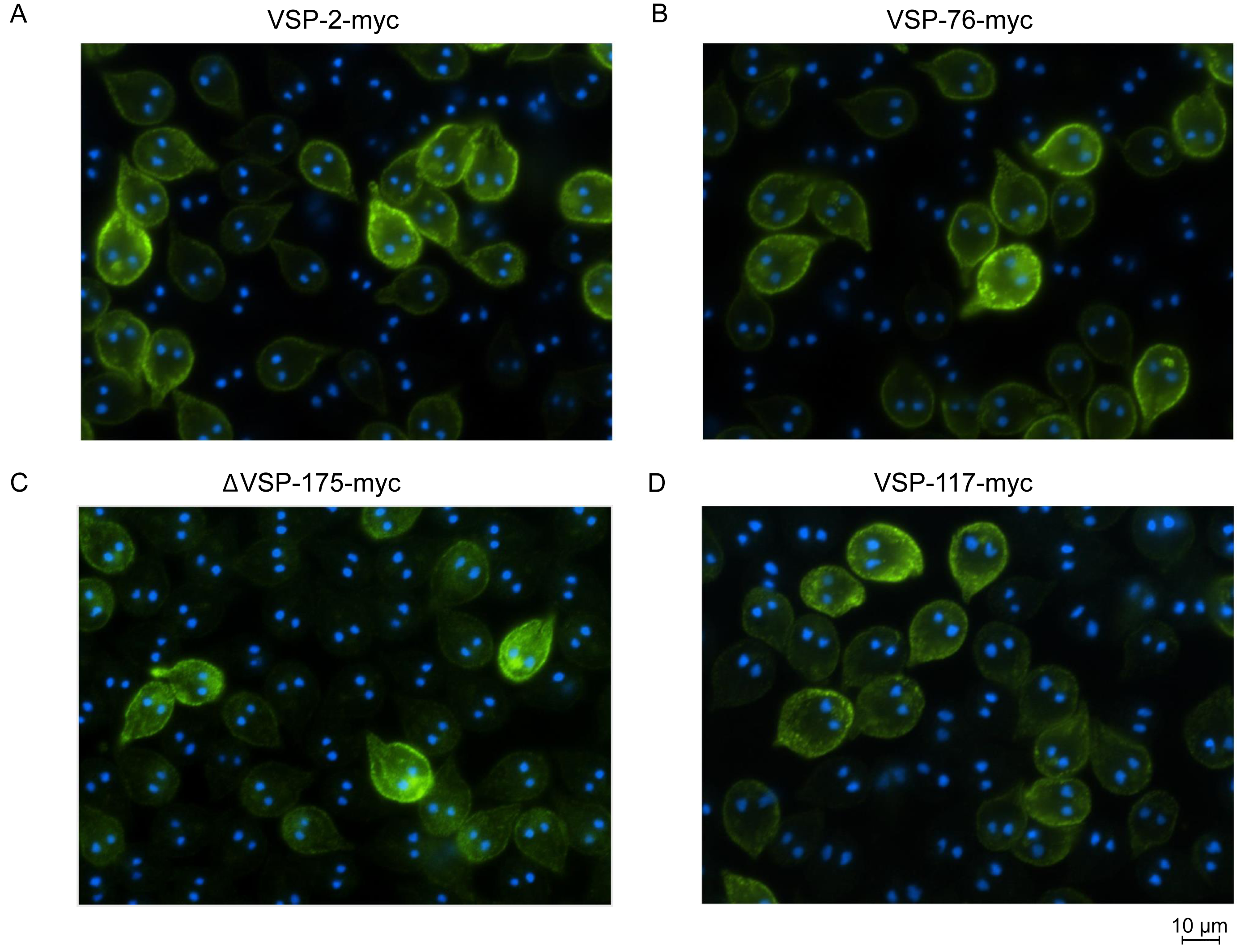

Supplement: Figure S1 — Immunofluorescent localization of C-terminal 3xmyc tagged VSPs (A, B, C, and D). All four 3xmyc tagged VSPs are expressed and localized to the membrane surface of Giardia trophozoites. (TIF) [file ppat.1003791.s001.tif]
