# Supplementary material for: The microRNAs in an Ancient Protist Repress the Variant-Specific Surface Protein Expression by Targeting the Entire Coding Sequence
Source: PLoS Pathog. 2014 Feb 27;10(2):e1003791. doi: 10.1371/journal.ppat.1003791 (PMC3937270; doi:10.1371/journal.ppat.1003791)
Supplement: Method S1 — Immunofluorescence assay. For detecting the C-terminal 3xmyc tagged VSP expression, indirect immuno-staining of nonpermeabilized cells were carried out as previously described (Li W, et al., in press). Briefly, the harvested Giardia cells were adhered to a poly-L-lysine coated cover slip (BD Biosciences). The cells were then fixed with 3% paraformaldehyde in NaPi (100 mM NaPi, pH 7.4) at room temperature, washed with NaPi, blocked with 0.2% gelatin in NaPi and immuno-stained using 1∶500 diluted anti-myc-FITC antibody (Invitrogen) in 0.2% gelatin in NaPi. The immuno-stained cells were examined using a Nikon TE2000E motorized inverted microscope equipped with 60×bright field and epifluorescence optics. Images were acquired with the NIS-Elements Advanced Research software (Nikon). (DOC) [file ppat.1003791.s002.doc]

**Supplemental Materials and Methods**

**Immunofluorescence assay**

For detecting the C-terminal 3xmyc tagged VSP expression, indirect immuno-staining of non-permeabilized cells were carried out as previously described (Li et al., 2013). Briefly, the harvested *Giardia* cells were adhered to a poly-L-lysine coated cover slip (BD Biosciences). The cells were then fixed with 3% paraformaldehyde in NaPi (100 mM NaPi, pH 7.4) at room temperature, washed with NaPi, blocked with 0.2% gelatin in NaPi and immuno-stained using 1:500 diluted anti-myc-FITC antibody (Invitrogen) in 0.2% gelatin in NaPi. The immuno-stained cells were examined using a Nikon TE2000E motorized inverted microscope equipped with 60×bright field and epifluorescence optics. Images were acquired with the NIS-Elements Advanced Research software (Nikon).

**References**

Li, W., Saraiya, A.A., and Wang, C.C. (2013). Experimental Verification of the Identity of Variant-Specific Surface Proteins in Giardia lamblia Trophozoites. MBio *4*.
